# Recent Advances in the Application of Metabolomics to Studies of Biogenic Volatile Organic Compounds (BVOC) Produced by Plant

**DOI:** 10.3390/metabo4030699

**Published:** 2014-08-21

**Authors:** Yoko Iijima

**Affiliations:** Nutrition and Life Science, Kanagawa Institute of Technology, 1030 Shimo-ogino, Atsugi, Kanagawa 243-0292, Japan; E-Mail: iijima@bio.kanagawa-it.ac.jp; Tel.: +81-46-206-0209; Fax: +81-46-291-3345

**Keywords:** plant-produced biogenic volatile organic compounds, BVOC, metabolomics, aroma compounds, herbivore-induced volatiles, GC-MS, LC-MS

## Abstract

In many plants, biogenic volatile organic compounds (BVOCs) are produced as specialized metabolites that contribute to the characteristics of each plant. The varieties and composition of BVOCs are chemically diverse by plant species and the circumstances in which the plants grow, and also influenced by herbivory damage and pathogen infection. Plant-produced BVOCs are receptive to many organisms, from microorganisms to human, as both airborne attractants and repellants. In addition, it is known that some BVOCs act as signals to prime a plant for the defense response in plant-to-plant communications. The compositional profiles of BVOCs can, thus, have profound influences in the physiological and ecological aspects of living organisms. Apart from that, some of them are commercially valuable as aroma/flavor compounds for human. Metabolomic technologies have recently revealed new insights in biological systems through metabolic dynamics. Here, the recent advances in metabolomics technologies focusing on plant-produced BVOC analyses are overviewed. Their application markedly improves our knowledge of the role of BVOCs in chemosystematics, ecological influences, and aroma research, as well as being useful to prove the biosynthetic mechanisms of BVOCs.

## 1. Introduction

Biogenic volatile organic compounds (BVOCs) are produced and emitted from various organisms [1]. The total amount of BVOCs emitted globally to the atmosphere is estimated to exceed 1 Pg per year [2]. These BVOCs include mainly plant-produced BVOCs, isoprene (44%), monoterpenes (11%), and other oxygenated carbon compounds, such as herbivore-induced volatiles and green leaf volatiles (22.5%) [2,3]. Their emission is easily affected by various abiotic global factors, UV strength, contents of ozone and CO_2_, drought conditions, eutrophication conditions, and warming [4]. The structures of BVOCs are complicated and chemically diverse, and more than 1700 volatile metabolites emitted from plant have been identified [5]. In many cases, the varieties of plant-produced BVOCs depend on the plant species, distinctive parts of the plants, or the circumstances under which the plants are growing [5,6,7,8]. The ecological roles of plant-produced BVOCs have been studied, and their roles are now known to include functions as defensive or attractive signals in interactions between plants and herbivores, pathogens, pollinators, and parasitoids, and more, the interactions between plants to plants by BVOCs are also observed [9,10,11,12,13,14]. The results of these studies indicate that the emission of BVOCs by plants is a useful communication tool. A particularly interesting point is that the structures of BVOCs that are synthesized for the above-mentioned interactions are sometimes unexpected and did not exist in the plants’ original condition (Table 1).

The BVOCs that are perceptible to humans are important as aroma and/or flavor compounds [15] (Table 1). Essential oils, which are concentrated cocktails of BVOCs obtained by distilled water or solvent extraction from various aromatic plants, have been produced since ancient times. The aroma strengths and characteristics of essential oils are determined by the profiles of the composed aroma compounds, which vary by genotypes and cultivation conditions [16]. Some aroma compounds show beneficial functions, such as antioxidant and antimicrobial activities, contributing to the compounds’ usefulness in food and cosmetics [17,18,19,20,21]. The composition of aroma compounds is thus a key factor in evaluations of the qualities of essential oils and agricultural products, such as flowers, vegetables, fruits, and herbs.

The common chemical properties of most VOCs are small molecules (up to 250 Da of molecular weight), and easy to be gaseous at ambient temperature, although some VOCs, such as ethanol and acetic acid, are soluble in water. The extraction and analysis methods used for VOCs are unique but may be matured in comparison to those for non-volatile compounds. VOC research, in the field of flavor/flagrance chemistry, has been conducted for many years, and the structures of many VOCs have been identified by synthetic chemical techniques. For example, standard technologies for VOC analyses are gas chromatography (GC) equipped with a flame ionization detector (GC-FID) or mass spectrometry (GC-MS). Many types of GC columns to separate various VOCs are available. In particular, the mass spectral databases for VOCs are better established compared to other natural non-volatile organic compounds; more than 600,000 compounds are searchable in mass spectrum libraries [22]. Regarding data analysis, chemometrics, a basic methodology for metabolomics, has been already accepted in the flavor chemistry field. Chemometrics has been used in evaluations of the aroma profiles of samples and in the differentiation of varieties and cultivation sites of materials and essential oils [23,24,25,26,27,28].

Metabolomics uses analytical methods to elucidate various biochemical events and dynamics in biological systems, based on the comprehensive characterization of the small-molecule metabolites [29,30]. This is achieved by systematically collecting multiple metabolic profiles and analyzing them comprehensively and simultaneously [31,32]. Metabolomics for volatile metabolites differs from flavor chemical analyses in that metabolomics emphasizes more biological aspects—especially physiological and environmental effects—in or by the synthesis of volatiles in plants. Metabolomics also examines the relationship between the synthesis of volatiles and their genetic backgrounds, and the metabolic networks between volatiles and non-volatile metabolites [1,33]. Thus, the metabolomics approach specifically for volatiles has recently been named “volatile metabolomics” or “volatilomics” [34,35]. Here, we introduce recently developed metabolomics techniques concerning BVOCs and their applications, focusing on the elucidation of volatile synthesis and its relationship to ecological and physiological events in plants.

**Table 1:** Main groups of plant-produced biogenic volatile organic compounds (BVOCs) and their functional characteristics.

Compound group	Typical BVOCs	Precursors (derived from)	Functional characteristics
Isoprenoids	Isoprene	IDP	Tolerance to sunlight- induced heating
Monoterpenes	*β*-Ocimene, *β*-Myrcene *α*-/*β*-Pinene, Limonene, Linalool, Geraniol	GDP	Harbivore-induced signal, Attractant to pollinator, Fragrance
Sesquiterpenes	*β*-Caryophyllene, *β*-Farnesene, Farnesol Nerolidol	FDP	Harbivore-induced signal, Antimicrobial activity
Homoterpenes	TMTT	(*E,E*)-Geranyllinalool	Harbivore-induced signal
	DMNT	(*E*)-Nerolidol	
Phenylpropenes	Eugenol, Methylchavicol	Phenylalanine	Aroma, Antioxidative activity, Antimicrobial activity
Benzenoids	Phenylethanol, Vanillin	Phenylalanine	Aroma, Flagrance
	Methyl salicylate	Phenylalanine/Isochorismate	Aroma, Harbivore-induced signal
Lipid derivatives	Hexanal, Hexenals, Hexanol, Hexenols, *(Z)*-3-Hexenyl acetate Methyl jasmonate	Fatty acids	Stress, damage, and herbivore-induced signal, Pathogen resistance
Aliphatic amino acid/lipid derivatives	Isomyl acetate, Isomyl alcohol	Leucine, Isoleucine	Fruit aroma
	Hexyl hexanoate	Acyl CoA	
S,N-containing	Isothiocyanates	Glucosinolates	Hervibore-induced signal, Antimicrobial activity, Flavor
	Disulfides, Trisulfides	*S*-alk(en)yl cysteine sulphoxides	

DMNT: 4,8-dimethylnona-l,3,7-triene; FDP: Farnesyl diphosphate; GDP: Geranyl diphosphate; IDP: Isopentenyl diphosphate; TMTT: 4,8,12-trimethyltrideca-1,3,7,11-tetraene.

## 2. The Procedures Used for Collecting and Extracting Plant-Produced BVOCs in Metabolome Analysis

Volatile synthesis in plants is known to be involved in both biotic and abiotic factors [33]. BVOCs can be (1) emitted by intact plants to the atmosphere; (2) stored in specialized tissues; and (3) synthesized after the disruption of tissues and cells. The procedures used to collect BVOCs are chosen in accord with the purpose of research, usually either by capturing one or more BVOCs from headspace gas or by extracting them with an organic solvent [36]. However, we have to recognize that the compositional profiles and concentration of BVOCs are differently provided by collecting procedures. Furthermore, some artifacts might be included in the BVOCs extracts from both procedures, because many of the volatiles are reactive.

**Figure 1:**
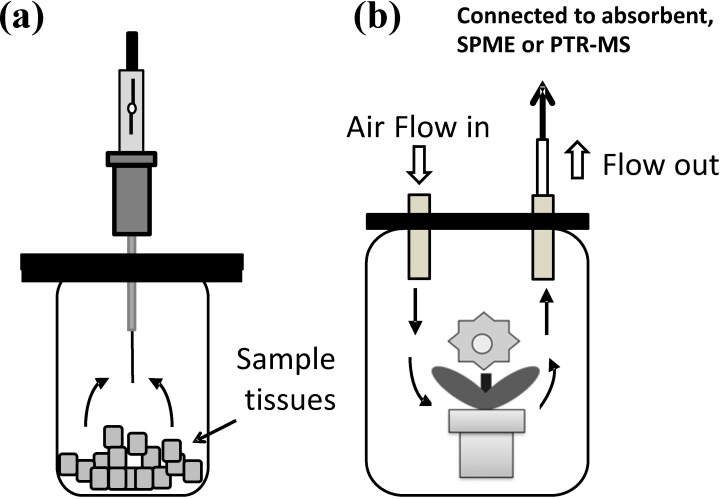
Typical headspace gas sampling systems. (**a**) Static headspace gas sampling with SPME; (**b**) Dynamic headspace gas sampling connected with absorbents, SPME or PTR-MS.

Headspace gas includes, for instance, emitted volatiles, such as flower scent and isoprene from trees, and the increasing aroma from ripening fruits, such as strawberry (*Fragaria × ananassa*) and melon (*Cucumis melo L.*). Synthesized BVOCs can freely cross the cell membrane and are, thus, released to the atmosphere [8,37]. Trapping headspace gas from plant samples is effective to obtain BVOCs without damage to the plants. Two types of methods, static and dynamic headspace sampling, are widely used for the collection of BVOCs [22,38] (Figure 1).

Static headspace sampling is more passive and classical than dynamic headspace, however, less efficient for trapping of BVOCs, since they are diluted in the chamber. Static headspace sampling is usually performed with solid-phase microextraction (SPME) fibers in the small volume vial (Figure 1), being popular for fruit volatile analysis. The advantage of this technique is that more vaporized metabolites are detected sensitively. Several types of absorbents for SPME, which are chemically different in polarity from each other are available to collect various BVOCs. The absorbed BVOCs are also thermally desorbed with ease at the injection port of a GC instrument. The advantages of this method are that it sensitively detects larger numbers of volatile and smaller molecule compounds without loss, and also can reduce the damage to GC-MS filaments. Automated SPME-GC under robotic control is available and allows high-throughput analyses with good reproducibility. SPME methods are thus widely used for metabolomics data collection by GC-MS. However, the extraction efficiencies of each compound differ by the SPME fiber used [39,40]. It is, thus, necessary to keep in mind that an SPME profile does not always show the real composition of emitted volatiles. Divinylbenzene/carboxen/poly(dimethylsiloxane) fibers were recently reported to be suitable for metabolomics studies as they provide the optimum extraction coverage and sensitivity for the widest range of analytes [40]. Since the compositional profiles of BVOCs are influenced by temperature, humidity, and their trapping period, the optimal condition for each plant sample must be carefully set up. To increase the capacity of VOC absorption, stir bar sorptive extraction (SBSE) and monolithic material sorptive extraction (MMSE) are also used for analyses of VOCs [41,42,43]. The surface area of SBSE and MMSE is larger than that of SPME fibers, enabling the adsorption of more VOCs compared to SPME. Thermal desorption can be used to perform the direct introduction of VOCs to a GC-MS system, although an additional thermal desorption unit is needed [44].

The dynamic headspace sampling technique is usually performed under air circulation by a flow through the system [38,45] (Figure 1b). The plant sample is put in an enclosed container with air-in and air-out valves. The outside/ambient air is made to constantly flow through the container by a circulation pump and vacuum pump, and BVOCs are collected by an absorbent trap connected to the output valve for the vacuum pump. As the absorbent, proapak-Q resin and Tenax^®^ resin are popular, and the absorbed BVOCs are extracted with a solvent such as dichloromethane, acetone, or diethyl ether [22]. Alternatively, thermal desorption is also acceptable for absorbent, and sometimes SPME method is used [44]. Furthermore, recent advances in volatile analysis enabled to detect BVOCs directly by proton transfer reaction-mass spectrometry (PTR-MS) (Figure 1b). The advantage of dynamic headspace analysis is that concentrated BVOCs are obtained under ambient conditions, because compositional changes in oxygen and carbon dioxide are avoided, and long-term experiments are possible with this technique. The dynamic headspace sampling technique is thus frequently used for the elucidation of volatile synthesis directly affected by environmental events, such as flowering, herbivore infestation, and other biotic or abiotic factors [38].

The solvent extraction procedure is sometimes used for VOCs from various samples. In plant materials, solvent extraction is performed to directly extract BVOCs stored in specific tissues, such as trichomes on the surfaces of plants and the flavedo in citrus fruits, and parts of aromatic plants and fruits [36]. Glandular trichomes in Lamiaceae and Solanaceae plants are well known to synthesize volatiles and store them in the subcuticular oil storage cavities on the top of trichomes [46,47]. The membrane of an oil storage cavity is easily disrupted by physical or chemical stimulation; most organic solvents easily penetrate the cellular membrane of the organism and break it. Organic solvent extraction may be, therefore, one of the easiest ways to quickly extract stored volatiles in plant, when a large number of independent samples are required for analysis of BVOCs. For example, mono/sesquiterpene profiles in tomato (*Solanum lycopersicum*) glandular trichomes were easily obtained from extraction by simply soaking the leaves in methyl t-butyl ether for 1 min [48]. Homogenized samples are sometimes used for volatile extraction, to improve the extraction efficiency [49,50]. However, here, we need to recognize that the solvent extracts include *de novo* synthesized BVOCs and non-/semi-volatile compounds, indicating that the compositional profiles from them are different from those from emitted BVOCs. Furthermore, contamination of non-volatile compounds sometimes disturbs to concentrate BVOC extracts and fouls the injection port of the GC instrument. In addition to the headspace sampling, SPME, and solvent extraction techniques, various steam distillation methods were classically developed and used to collect BVOCs [1,36].

Several technologies for the simple and sensitive detection of VOCs without extraction or adsorption were recently developed. PTR-MS enables the analysis of VOCs in real-time by using proton transfer reagent ions such as H_3_O^+^ ions. This is expected to be very effective for analyzing sequential changes in the composition of VOCs. Indeed, differences in the emission patterns of the three green leaf volatiles *(Z)*-3-hexenal, *(Z)*-3-hexen-1-ol and *(Z)*-3-hexen-1-yl acetate after wounding of *Arabidopsis* leaves were successfully monitored by PTR-MS [51]. Direct analysis in real time ion-source mass spectrometry (DART-MS) can directly ionize the metabolites in gas, liquid and solid samples at atmospheric pressure and detect them by MS [52,53,54]. Block * et al.* [55] used DART-MS to directly monitor the sequential metabolic changes of sulfur compounds after crushing garlic (*Allium sativum*). The properties of PTR-MS and DART-MS in simple, high-throughput, and sequential analyses, are expected in applications for metabolomics.

Imaging mass spectrometry (IMS) using matrix-assisted laser desorption/ionization (MALDI)-MS, atmospheric pressure ion-source chamber for laser desorption/ionization (AP-LDI)-MS, and nano-particle laser desorption/ionization (nano-PALDI)-MS enables the visualization of metabolites in tissues and cells. IMS is very useful to clarify the distribution of the target molecules. Various specialized metabolites stored in the trichomes of wild tomato were recently successfully detected by IMS [56]. As for plant volatiles, the MS ions of monoterpene, diterpene and 6-gingerol were visualized in the tissue of ginger (*Zingiber officinale*) rhizome by (AP-LDI)-MS microscopy [57]. Compared to the studies of the biosynthesis of plant volatiles, the distribution and transportation of these small molecules in plant tissues, * i.e.*, how these small nonpolar compounds are secreted to the specialized tissues or released to the outside, are not well understood [58]. Therefore, sequential mass images of volatiles in the tissues are expected to help clarify the movement of volatiles among tissues.

## 3. Data Mining from Multiple Volatile Profiles by Metabolomics Techniques

In metabolomics, the techniques used for the data analysis to handle vast data files are very important, as is the sensitive and comprehensive detection of metabolites. Targeted and non-targeted metabolite data analyses and sometimes wide-target metabolite analyses are frequently performed in metabolomics [59,60]. A targeted metabolite analysis focuses on “identified” metabolites whose chemical structures are clear and for which the standard compounds are available. This analysis includes the quantification of each metabolite, contributing to our understanding of the biochemical dynamics and metabolic relationships among the metabolites. In a non-targeted metabolite analysis, all detected peaks including those of unknown compounds are counted comprehensively and then applied to a multivariate analysis such as a principal component analysis (PCA) or partial least squares-discriminant analysis (PLS-DA) analysis. The significant metabolite peaks are then filtered, and their structures are chemically investigated. This is a valuable technique for screening novel biomarkers and for finding unexpected metabolites. However, in non-targeted analyses, the frequently detected “unknown” peaks make a detailed analysis difficult, and this has prompted the development of various MS-based peak annotation/identification procedures and metabolite databases [60,61].

In the research for plant volatiles, targeted metabolite analyses are more conventional, because many standard mass spectra for VOCs have accumulated in MS databases and many targeted BVOC profiles of various plant species have been reported. The first volatile analysis termed “metabolomics” was reported for volatile profiling in petunia (*Petunia x hybrida*) flowers [62]. Regarding non-targeted metabolite analyses for volatiles, a successful new strategy providing an unbiased comparative multivariate analysis was first reported for large-scale data-mining, using 94 genotypes of tomato (*S. lycopersicum*) fruits [63]. In that analysis, 322 VOCs including unknown compounds were distinguishable, and they were clustered into several groups of biochemically similar compounds in a hierarchical cluster analysis. This innovative analytical strategy provided clues to the biosynthesis and function of unknown metabolites. More recently, non-targeted analyses concerning BVOCs have been used to elucidate the characteristics of wine and carrot (*Daucus carota*) flavors [64,65] , the emission of herbivore-induced VOCs from wild tobacco (*Nicotiana attenuata*) [66], the biodiversity of pepper (*Capsicum sp.*) [67], and more. The development of bioinformatics tools focused on plant volatile data has continued to advance [68], and a mass-based automated peak annotation and database system has been developed; it is known as vocBinBase [69].

## 4. Application of BVOC Metabolome Analyses to Functional Studies of Volatiles

### 4.1. The Use of BVOC Metabolomics in Chemotaxonomic Studies

Chemotaxonomy, also called chemosystematics, aims to classify and identify certain organisms according to their metabolic compositions, and to evaluate the organisms’ biological diversity and evolution in light of their metabolite diversity. In many cases of plant samples, secondary metabolites are targeted for analysis because their biosynthesis is usually specific to certain plant species [70]. In the typical chemotaxonomic study, varieties of a certain plant species are collected and their metabolic profiles are obtained. After the quantitative data of the targeted metabolite are obtained, a further multivariate analysis, such as a hierarchical clustering analysis (HCA) or PCA, is performed to evaluate similarities among the samples and to identify markers of taxonomic differences.

Thus, the profiling of essential oil, which is constituted of BVOCs was frequently performed in chemotaxonomic studies of medicinal plants and aromatic plants before the concept of metabolomics concept had arisen [71,72,73]. Terpenes (mono-, and sesqui-) are the most valuable target markers in many plants [74,75,76]. The relationships between the chemotypic diversity in essential oils and their genetic diversity were investigated in *Salvia fruticosa* [77], *Rosmarinus officinalis* [78], and *Ocimum gratissimum* [79]. This approach in a chemotaxonomic study is very similar to the metabolomics approach. Recent advances in metabolomics and genomics technologies will help accelerate the detailed analyses of the correlations between chemical composition and genetic diversity.

### 4.2. Application of VOC Metabolomics to the Characterization of the Biosynthesis of Targeted Volatiles

**Figure 2:**
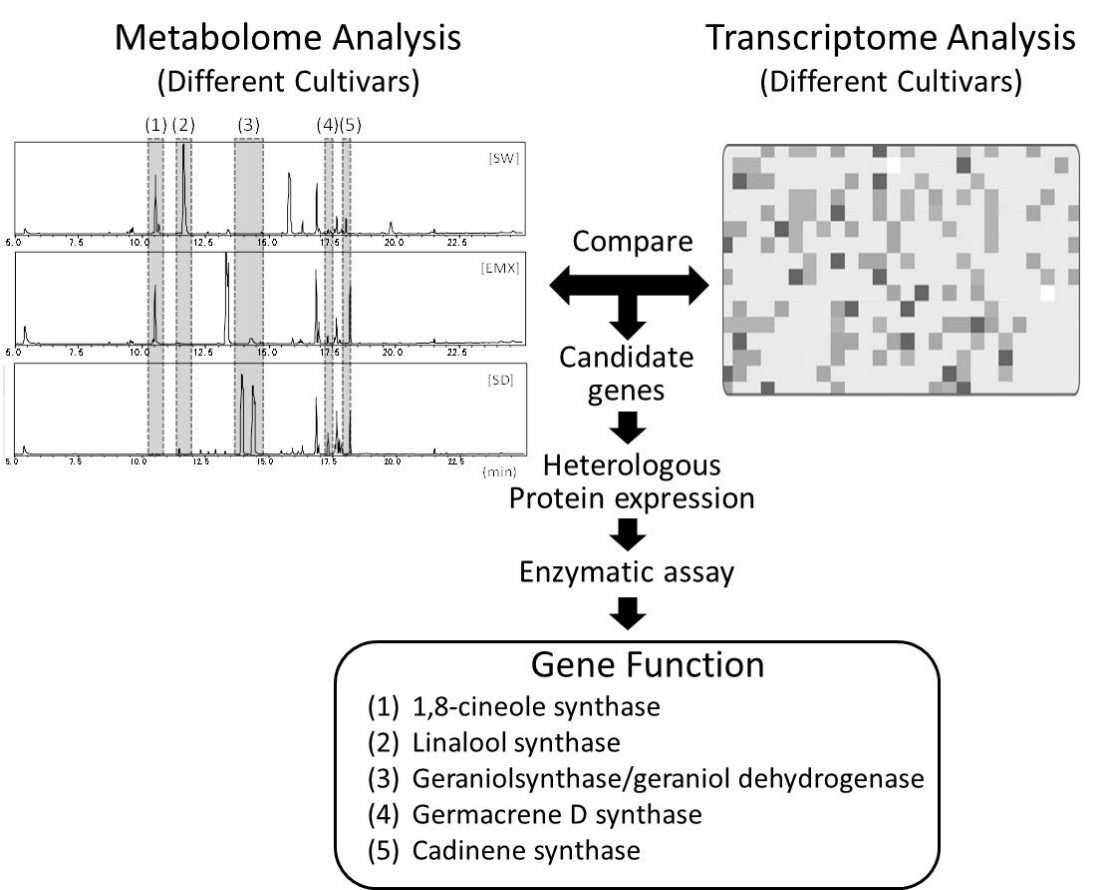
Metabolomics-driven screening of gene functions for synthesis of BVOCs. This is an example of finding of terpene synthase gene functions involved in aroma of Sweet basil (*Ocimum basilicum*) [50,84]. Integration of metabolome and transcriptome data facilitated the elucidation of gene function.

Metabolomics also has great merit in combination with other “omics” data, such as genomics, transcriptomics, and proteomics. In particular, the correlation of the profiles of gene expressions at the mRNA level and metabolite accumulation will elucidate the gene functions in the biosynthesis of various specialized metabolites [31,60,80,81]. This strategy, called functional genomics, has been used frequently in comparative analyses of plant volatiles, as well as other specialized metabolites. The characterization of gene functions involved in the biosynthesis of specific volatiles has been successfully clarified in various plants (in both model plants and non-model plants) by performing comparative analyses of samples prepared from different varieties/cultivars and different mutant lines, by different physiological conditions such as developmental stages, and in light of the effects of herbivore infestation [8,82,83] (Figure 2).

To elucidate the genetic bases of specialized metabolite biosynthesis, its regulation and metabolite diversity, quantitative trait loci (QTL) mapping of various metabolites was carried out in plant materials, mainly in model plants such as *Arabidopsis thaliana* and tomato (*S. lycopersicum*) [85]. Here, metabolomics is an indispensable analytical tool for obtaining extensive data from a variety of crossed lines and handling them for QTL analysis [86,87,88,89]. The QTL mapping of aroma compounds was conducted for apple (*Malus sp.*, “Discovery” *×* “Prima”) [90], tomato (*S. lycopersicum* × *S. pennellii*) [91,92,93], *Sorghum bicolor* [94], strawberry (*Fragaria × ananassa*) [95], and petunia (*Petunia axillaris* × *Petunia exserta*) [96]. Insights provided by metabolite QTL-assisted genome sequencing successfully characterized novel biosynthetic enzymes involved in aroma compounds such as phenylethanol [97], aliphatic alcohol/aldehydes/esters [98], and mono-/sesqui-terpenes [99] from tomato introgression lines (*S. lycopersicum* × *S. pennellii*).

### 4.3. Application of Metabolomics to Ecological Studies Concerning Volatiles

Metabolomics can be used to evaluate a plant’s metabolic changes in response to environmental circumstances (*i.e.*, abiotic conditions), e.g., drought conditions, nutrient availability and temperature alteration [100]. Biotic interactions between a plant and other organisms also affect the metabolic dynamics [101,102]. These findings indicate that an ecological condition can induce marked metabolic changes in both the central metabolism and specialized metabolism [14]. Metabolomics has, thus, been received as a powerful analytical methodology in ecological studies [37,100].

BVOCs are also a type of metabolite group influenced by ecological conditions. Many studies indicated that the emission of BVOCs from plant occurs as significant cues, signals, or defense responses to wounding, herbivore infestation, pathogen infection, and pollination [103]. The emitted BVOCs are also known to act as a plant-to-plant communication tool [104]. Interestingly, the composition of emitted BVOCs is frequently influenced by treatment procedures and plant species. In tomato (*S. lycopersicum*), the BVOC emission was changed by four types of damage treatments: tomato psyllid nymphs (*Bactericera cockerelli*), cabbage looper caterpillars (*Trichoplusia ni* Hübner), fall Armyworm caterpillars (*Spodoptera frugiperda* Smith), and mechanical damage [105]. In cereal crops (wheat, oat, and barley), mechanical injury, beetle (*Oulema spp.*) herbivory, and fungal infection (*Fusarium spp.*), induced different ratios of BVOC blends, and, moreover, their composition is different by species [106]. Thus, metabolome data can reveal the differentiation of volatile metabolites by ecological conditions. In many studies, green leaf volatiles and specific terpenoids were found as herbivore-induced compounds by comparative metabolic analyses of damaged and undamaged plants [66,107,108,109] (Table 1).

The integration of metabolomics with transcriptomics facilitated the elucidation of the biosynthesis of herbivore- and pathogen-induced metabolites, the defense system evoked by damage, and interactions with other organisms [100]. In tomato plants (*S. lycopersicum)*, the influence of a spider mite (*Tetranychus urticae*) infestation was investigated using a combined metabolome and transcriptome analysis. It was observed that the emission of volatiles, which were preferred by predatory mites occurred several days later than the spider mite-induced defense response, suggesting that volatile production is an indirect defense system that complements the direct defense response against spider mites [110]. In another study, comparative transcriptome and metabolome profiles of pest (*Tortrix viridana* L.) herbivore-resistant and -susceptible oak (Quercus robur L.) were obtained, and they indicated that the defense system for each cultivar of oak differs at both the transcription and metabolite levels [111]. Metabolomics for BVOCs is a reliable technique to investigate physiological events in a plant in response to its circumstances.

### 4.4. Application of VOC Metabolomics to Plant Breeding and Quality Evaluations for Commercial Demand

Metabolomics has contributed to the breeding and biotechnology of crops, with the goal of improving food qualities [112,113], and has been accepted in the fields of horticulture, agriculture and food science [114,115,116]. In many fruits, vegetables, and herbs, controlling of their aroma and flavor characteristics is one of the significant factors related to improving their qualities [117,118,119,120]. Further advances in the quality of food products will require the consideration of not only genotypes/cultivars, but also cultivation conditions and preharvest/postharvest handling.

For these purposes, the most extensively investigated food thus far is tomato (*S. lycopersicum*) and its products [121]. Over 400 volatiles have been identified in fresh tomato and tomato products [122,123]. The differences in sensory characters and BVOC profiles among tomato cultivars and mutants have been examined [63,124,125,126,127]. To identify the compounds that affect consumers’ tomato preferences, Berna *et al.* [124] investigated the correlation of aroma profile data with a consumer-preference test using various commercially available tomato cultivars. They concluded that consumer preference is strongly explained by 3-methylbutanol, *(E)*-2-hexenaol and *(Z)*-3-hexenol. Cebolla-Cornejo *et al.* [126] reported that the composition of aroma compounds in a tomato is more affected by genotype than environmental conditions. Tomato is a model plant for climacteric fruits, which produce ethylene and autocatalytically percept it for ripening [128,129]. This ripening system regulates various gene expressions controlling fruit softening and the accumulation of various metabolites, such as sugars, acids, amino acids, carotenoids, and aroma compounds [130]. In an investigation of the ripening system in tomato, some mutant lines related to ethylene synthesis or ethylene perception were used for a comparative analysis [131]. Regarding aroma biosynthesis in tomato, a comparative BVOC metabolome analysis within a ripening mutant (*rin*: ripening inhibitor, *nor*: non-ripening, *Cnr*: Colourless nonripening, *Nr*: Never-ripe, *hp1*: high pigment mutant 1) indicated that lipid-derived BVOCs such as *(Z)*-3-hexenal, (*E*)-2-hexenal, and hexanal are responsible for the differentiation of unripe mutant lines (*rin*, *nor*, *Cnr* and *Nr*) from control and *hp1* [132]. It was also suggested that the transcription level of *TomloxC*, a kind of lipoxygenases, is involved in these compositional differences [132].

Other than tomato, insights into screening quality markers provided by metabolomics-driven BVOC profiles were reported for melon (*Cucumis melo*) [133,134], apple (*Malus pumila*) [135,136], strawberry (*Fragaria × ananassa*) [137], and pepper (*Capsicum sp.*) [67]. Moreover, recent studies directly emphasized the relationship between some specific aroma compositions and sensory perceptions for consumer preference, for not only food material but also processed food, such as wine [64] and beer [138].

## 5. Interrelationships between Volatile and Non-Volatile Metabolites Elucidated by Multiplatform Metabolomics Analyses

The structures of plant-produced BVOCs are categorized mainly into groups of mono-/sesqui-terpenoids, benzenoids/phenolics, amino acid derivatives and lipid degradation/derivatives. In many cases, they are terminal products in the entire metabolic pathway in plants, although terpenes are well known to be sometimes modified to semi/non-volatile compounds, for example, by oxygenation in resin ducts of conifer [139]. The amounts of the BVOCs produced are much less than those of other precursor metabolites in the central metabolism. In addition, many VOCs are relatively hydrophobic, whereas most of their precursors and intermediates are highly polar and non-volatile. It is, thus, necessary to view the entirety of the metabolic changes including non-volatile fraction in order to clarify the biosynthesis pathways of BVOCs. Toward this end, metabolome analyses incorporating isotope-labeled precursors are effective for evaluating metabolite flux [140].

Notably, stable isotope-labeled compounds are detected as having m/z values that are different from those of endogenous metabolites, indicating that a GC- or LC-MS analysis is useful to follow-up their metabolic flow. In petunia (*Petunia hybrida*) flower, deuterium-labeled phenylalanine was used to investigate the biosynthetic pathway of benzenoid volatiles, and a combined analysis by GC-MS (for volatiles) and LC-MS (for non-volatiles) successfully identified the pathway of benzenoid synthesis [141].

The emission of BVOCs occurs under various biotic and abiotic conditions. The BVOCs are released through the membranes of the epidermal tissues, stomata, or other specialized structures, such as trichomes and osmophores [37]. Some plants emit airborne BVOCs that are suggested to be taken up into plants at the plant surface via stomatal conductance or cuticle diffusion [104]. In such plant-plant communications, the hormone-like BVOCs methylsalicylate, methyljasmonate, green-leaf volatiles (C6-aldehydes and alcohols) and ethylene, are proposed as communication signals [104].

**Figure 3:**
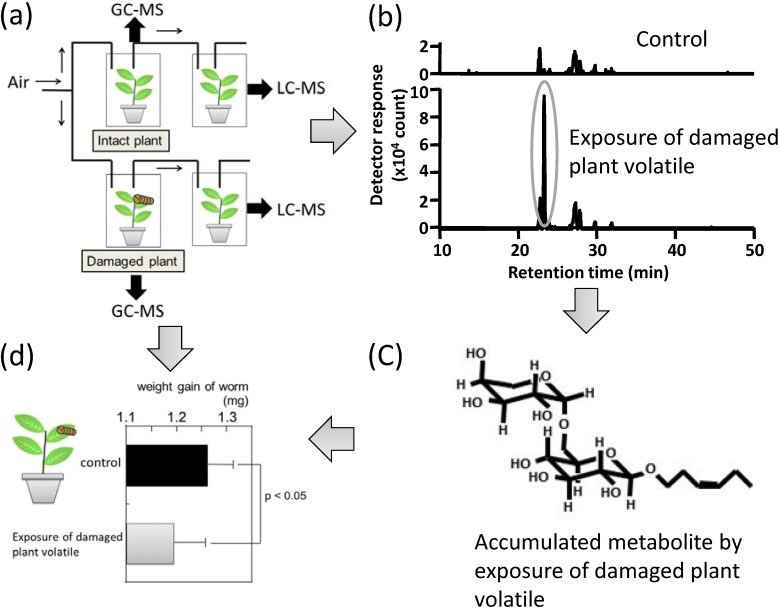
Multiplatform metabolic profiling identified the mechanism of plant-plant communication by green leaf volatiles. (**a**) Airflow setup for volatile exposure. Intact tomato (*S. lycopersicum*) plant and the plant damaged by cutworm (*S. litura*) were prepared; (**b**) Comparative metabolic profiling of exposed plants by LC-MS analysis. The leaves after exposure of damaged plant volatiles accumulated a specific metabolite; (**c**) Determination of synthesized metabolite. The structure of it was determined as (*Z*)-3-hexenyl vicianoside; (**d**) Weight gains of cutworm (*S. litura*) on exposed leaves were less than those on control leaves.

We were recently the first group to demonstrate that *(Z)*-3-hexenol, which is a main BVOC emitted from tomato (*S. lycopersicum*) plants after an infestation with cutworms (*Spodoptera litura*) , was received into a neighboring intact plant (Figure 3). In addition, this received plant showed defense to be infested by this worm. Our non-targeted metabolome analysis of leaves of the receiving plant by GC-MS and LC-MS showed that the incorporated *(Z)*-3-hexenol was accumulated as a glycoside conjugation (vicianoside) to acquire defense [142]. This finding indicates that incorporated *(Z)*-3-hexenol is directly metabolized in the leaf tissues. Such accumulations of metabolized BVOCs, mainly glycosylated alcoholic BVOCs, were found in several plants, and they are recognized as important aroma precursors, e.g., in rose (*Rosa damascene*) petal [143,144], tea (*Camellia sinensis*) leaf [145], wine grapes (*Vitis spp.*) [146], and tomato (*S. lycopersicum*) fruit [147]. Since volatile alcohols are liberated by glycosidase derived from endogenous or microbial enzymes [148], evaluations of the amounts of glycosylated BVOCs could provide a valuable index of flower and food quality. Tikunov * et al.* [149] performed a non-targeted fusion metabolome analysis with GC-MS for volatiles and LC-MS for non-volatiles in different tomato (*S. lycopersicum*) genotypes. They found that the emission of phenolic aroma compounds is regulated essentially by the cleavage of their disaccharide derived from cell disruption during tomato ripening, whereas the low phenolic aroma genotypes led to the conjugation of more complicated forms, such as trisaccharides, and malonylated disaccharides. These results indicate the strong potential that multiplatform metabolomics can provide novel findings concerning the regulation of BVOC synthesis and the effects of BVOCs on other physiological and ecological functions.

## 6. Perspective

Recent BVOC analysis techniques have improved, especially regarding the monitoring of BVOCs’ spatial and temporal changes. Although the sensitivity and comprehensiveness of the analyses still need improving, these techniques will contribute to the detailed understanding of the metabolic dynamics of BVOCs. In this review, we focused on metabolomics for the production of BVOCs from plant and their profiles, with only a brief mention of the indirect metabolic changes induced by BVOC reception. Plant defense systems often operate through a jasmonic acid and salicylic acid pathway, affecting the metabolite balance of central metabolites [150]. To systematically identify the effects of VOCs on plants, more extensive metabolome analyses are needed, including those examining central metabolites, BVOCs and other non-volatile specialized metabolites. Most BVOCs exist constitutively on the earth. Recent interest in and research into the biological roles of BVOCs have highlighted the potential usefulness of BVOCs in ecosystems. The elucidation of the regulation of BVOC synthesis and the effective mechanisms of each BVOC would also contribute to the metabolic engineering of volatiles [151].

Metabolome data can be applied to cross-analyses with other related quantitative data concerning phyisiological status and olfactometry than metabolites. For instance, in food materials, the determination of the relationship between quantitative sensory evaluation data and metabolome data will be effective for screening the contributing metabolites, and for the prediction of preference models [152]. Thus, improvements in the technologies used for accurate quantitative measurements of physiological and olfactory effects, will help identify the new roles of BVOCs.
